# Crosstalk between NSL Histone Acetyltransferase and MLL/SET Complexes: NSL Complex Functions in Promoting Histone H3K4 Di-Methylation Activity by MLL/SET Complexes

**DOI:** 10.1371/journal.pgen.1003940

**Published:** 2013-11-14

**Authors:** Xiaoming Zhao, Jiaming Su, Fei Wang, Da Liu, Jian Ding, Yang Yang, Joan W. Conaway, Ronald C. Conaway, Lingling Cao, Donglu Wu, Min Wu, Yong Cai, Jingji Jin

**Affiliations:** 1 School of Life Sciences, Jilin University, Changchun, Jilin, China; 2 Stowers Institute for Medical Research, Kansas City, Missouri, United States of America; 3 Department of Biochemistry and Molecular Biology, Kansas University Medical Center, Kansas City, Kansas, United States of America; 4 College of Life Sciences, Wuhan University, Wuhan, Hubei, China; 5 National Engineering Laboratory for AIDS Vaccine, School of Life Sciences, Jilin University, Changchun, Jilin, China; Emory University, United States of America

## Abstract

hMOF (MYST1), a histone acetyltransferase (HAT), forms at least two distinct multiprotein complexes in human cells. The male specific lethal (MSL) HAT complex plays a key role in dosage compensation in *Drosophila* and is responsible for histone H4K16ac *in vivo*. We and others previously described a second hMOF-containing HAT complex, the non-specific lethal (NSL) HAT complex. The NSL complex has a broader substrate specificity, can acetylate H4 on K16, K5, and K8. The WD (tryptophan-aspartate) repeat domain 5 (WDR5) and host cell factor 1 (HCF1) are shared among members of the MLL/SET (mixed-lineage leukemia/set-domain containing) family of histone H3K4 methyltransferase complexes. The presence of these shared subunits raises the possibility that there are functional links between these complexes and the histone modifications they catalyze; however, the degree to which NSL and MLL/SET influence one another's activities remains unclear. Here, we present evidence from biochemical assays and knockdown/overexpression approaches arguing that the NSL HAT promotes histone H3K4me2 by MLL/SET complexes by an acetylation-dependent mechanism. In genomic experiments, we identified a set of genes including ANKRD2, that are affected by knockdown of both NSL and MLL/SET subunits, suggested they are co-regulated by NSL and MLL/SET complexes. In ChIP assays, we observe that depletion of the NSL subunits hMOF or NSL1 resulted in a significant reduction of both H4K16ac and H3K4me2 in the vicinity of the ANKRD2 transcriptional start site proximal region. However, depletion of RbBP5 (a core component of MLL/SET complexes) only reduced H3K4me2 marks, but not H4K16ac in the same region of ANKRD2, consistent with the idea that NSL acts upstream of MLL/SET to regulate H3K4me2 at certain promoters, suggesting coordination between NSL and MLL/SET complexes is involved in transcriptional regulation of certain genes. Taken together, our results suggest a crosstalk between the NSL and MLL/SET complexes in cells.

## Author Summary

Covalent modification of N-terminal tails of histone proteins is accomplished by a variety of chromatin modifying complexes. These complexes catalyze at least eight distinct types of histone modifications including acetylation, methylation, phosphorylation, and ubiquitination. Histone modifications may act alone or in a coordinated manner to activate or repress chromosomal processes. For example, a particular histone modification may recruit or activate chromatin modifying complexes that generate a different histone modification. Coordination between hMOF-mediated histone H4K16 acetylation and other histone modifications has been reported by several research groups. The presence of subunits shared between the hMOF-containing NSL and MLL/SET family complexes suggests there may be functional links between two complexes. Consistent with this idea, we identified a set of genes that are co-regulated by the NSL and MLL/SET complexes. Both *in vitro* and *in vivo* experimental approaches provide evidence that the NSL HAT functions in promoting histone H3K4 di-methylation activity by MLL/SET complexes. Interestingly crosstalk between hMOF/NSL HAT and MLL/SET HMT activity seems to be unidirectional since there we detected no effect of MLL/SET activity on NSL HAT, either *in vitro* or in cells.

## Introduction

The precise organization of chromatin is critical for many cellular processes including gene transcription, recombination, DNA replication and damage repair [1]. Changes of chromatin structures are mainly regulated by epigenetic regulations such as ATP-dependent remodeling of nucleosomes, the incorporation of variants histones into nucleosomes and post-translational modifications of histones [2]. Post-translational modifications of the N-terminal tails of histones including acetylation, methylation, phosphorylation, ubiquitination and ADP-ribosylation may act alone or in a coordinated manner to facilitate or repress chromatin-mediated processes [3]–[5]. Crosstalk between different modifications may be accomplished by a number of mechanisms. For example, an initial histone modification may trigger increased activity of a histone-modifying enzyme. Alternatively, one histone and its modifications affect the modification of a different histone [6]. Thus, acetylation of histone H3 on lysine 18 and lysine 23 promotes the methylation of argine 17 by the CARM1 (coactivator-associated arginine methyltransferase 1) methyltransferase, resulting in activation of estrogen-responsive genes [7]. Also, methylation of H3K4 by COMPASS (complex of proteins associated with Set1) and of H3K79 by Dot1 is totally dependent upon the ubiquitylation of H2BK123 by Rad6/Bre1 in *Saccharomyces cerevisiae* [8].

hMOF (MYST1), a member of the MYST family of histone acetyltransferases (HATs), is the human ortholog of *Drosophila* male absent on the first (dMOF) protein [9]. Depletion of hMOF in cells leads to genomic instability, spontaneous chromosomal aberrations, cell cycle defects, reduced transcription of certain genes, and defective DNA damage repair and early embryonic lethality [10]–[13]. Moreover, the role of MOF in the DNA damage response is conserved in mammalian cells and *Drosophila* [14]. Genome-wide analysis demonstrates that MOF is not only involved in the onset of dosage compensation, but also acts as a regulator of gene expression throughout the *Drosophila* genome, suggesting the functional diversity of MOF [15]. Recent biochemical purifications have revealed that MOF forms at least two distinct multi-protein complexes, MSL and NSL, in *Drosophila* and mammalian cells [16]–[18]. Although the functions of MSL and NSL complexes in human cells are not entirely clear, both complexes can acetylate histone H4 at lysine 16 (H4K16), suggesting the importance of acetylation of H4K16 in cells [19]–[20]. Besides H4K16, NSL complex is also able to acetylate other histone H4 lysines, such as H4K5 and H4K8 [17]. Intriguingly, NSL complex appears to be involved in more global transcription regulation as it has been found to bind to a subset of active promoters and contribute to housekeeping gene expression in *Drosophila* [21]–[23]. It is noteworthy that the NSL complex shares subunits with other chromatin regulating complexes. The MCRS1 (Microspherule Protein 1) protein is a shared subunit between the NSL complex and the Ino80 chromatin remodeling complex [24]. The WDR5 protein is a subunit not only of the NSL complex, but also of the MLL/SET-containing histone H3K4 methyltransferase complexes [25] and of the ATAC histone acetyltransferase complex [26]. The presence of these cross-shared subunits suggests functional links between these complexes and the histone modifications they catalyze.

Coordination between hMOF-mediated histone H4K16 acetylation and other histone modifications has been reported by several research groups. In the response of 293 cells to serum stimulation, the phosphorylation of H3S10 is the trigger for H3K9 and H4K16 acetylation, resulting in transcription activation and elongation [27]. In addition, hMOF-mediated H4K16ac and SUV420-H2-mediated H4K20me3 antagonistically control gene expression by regulating Pol II promoter-proximal pausing [28]. Given that WDR5 is part of the MLL/SET-containing methyltransferases and hMOF/NSL acetyltransferase, the interaction between H3K4me and H4K16/K5/K8ac is speculated. FOXP3, an X-linked suppressor of autoimmune disease and cancers, increases both H4K16 acetylation and H3K4 trimethylation at the FOXP3-associated chromatins of multiple FOXP3-activated genes by recruiting MOF and displacing histone H3K4 demethylase PLU-1 [29]. All the above investigations strongly suggest that the coordination between hMOF-mediated H4K16 acetylation and other histone modifications is involved in certain gene transcriptional activation. However, the precise cooperative mechanism between different histone modifications remains unclear.

In an effort to resolve the coordination role in the activities between hMOF-mediated H4K16 acetylation and MLL/SET-mediated H3K4 methylation, we have carried out systematic biochemical and molecular biological analysis of human MOF/NSL HAT and MLL/SET complexes. As we describe below, our findings demonstrate a new regulatory pathway, that the hMOF/NSL-mediated histone H4K16 acetylation facilitates histone H3K4 di-methylation by MLL/SET complexes. This functional interaction leads to a coordinative regulation of certain downstream target genes, such as ANKRD2.

## Results

### Crosstalk between WDR5-containing HAT and HMT in Flag-WDR5 complex

**Figure 1:**
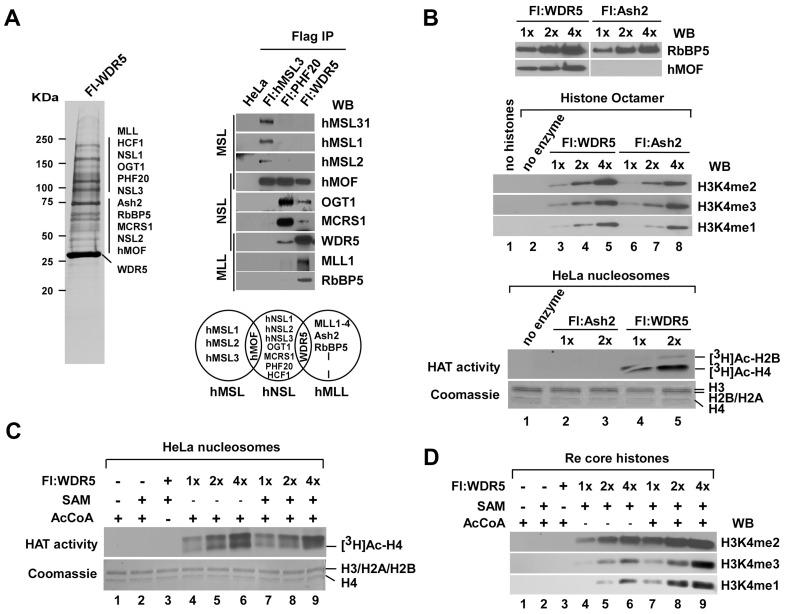
HAT activity facilitates histone H3K4 methylation by WDR5-containing complex *in vitro*. **A.** WDR5 is a shared subunit between HAT NSL and HMT MLL/SET complexes. Anti-Flag agarose eluate from a Flag-WDR5 stable cell line was analyzed by SDS-PAGE, and proteins were visualized by silver staining (left). Equal amounts of anti-Flag agarose eluates from Flag-PHF20 (a subunit of NSL complex), Flag-MSL3L1 (a subunit of MSL complex) and Flag-WDR5 (a shared subunit between NSL and MLL/SET complexes) were fractionated by SDS-PAGE, and proteins were identified with Western blot using the indicated antibodies (right). **B.** Immunoaffinity purified WDR5-containing complex possesses a dual enzymatic activity. hMOF and RbBP5 protein from Flag-WDR5 and Flag-Ash2- (a subunit of MLL/SET complexes) expressing cells were detected with Western blotting using the indicated antibodies (top). Relatively equal amounts of RbBP5 proteins in WDR5- and Ash2-containing complexes were used in HMT assays. Modified residues at H3K4 were detected with methylation specific antibodies (middle). HAT assays were performed with 0.5 µCi of [^3^H] AcCoA and 3 µg HeLa nucleosomes. Histone proteins were visualized by Coomassie R-250 blue stain and radioactively labeled proteins were visualized by autoradiography (bottom). **C.** HAT activity of the WDR5-containing complex is not changed in the presence or absence of SAM *in vitro*. **D.** HMT activity of the WDR5-containing complex is increased in an AcCoA-dependent manner. HMT assays were performed with recombinant core histones in the presence or absence of AcCoA *in vitro*.

WDR5 has been shown to be a shared subunit of multi-complexes such as histone acetyltransferases and histone methyltransferases [17], [25]–[26]. To elucidate interactions between the WDR5-containing HAT and HMT, we previously generated a human cell line stably expressing Flag-WDR5, purified Flag-WDR5-associating proteins by anti-Flag immunoaffinity chromatography, and analyzed them by MudPIT mass spectrometry and Western blotting. MudPIT analyses of Flag-WDR5-associating proteins identified previously defined subunits of human MLL/SET histone methyltransferase complexes and hMOF-containing NSL histone acetyltransferase complex [17]. These findings were confirmed by immunoblot using available antibodies. As shown in Figure 1A, anti-Flag eluates from Flag-MSL3L1 (a specific subunit for MSL complex), Flag-PHF20 (a specific subunit for NSL complex) and Flag-WDR5 expressing cells were fractionated by SDS-PAGE and analyzed by western blotting. The results revealed that the subunits of both MLL/SET and hMOF-containing NSL HAT complexes could be detected in anti-Flag agarose eluates from Flag-WDR5 expressing cells, consistent with previous evidence that WDR5 complex is associated with at least two multi-protein complexes: MLL/SET HMT and NSL HAT [17].

To characterize the enzymatic activity(s) that copurified with Flag-WDR5-containing complexes, *in vitro* HAT and HMT assays were performed. As predicted, Flag-WDR5 is associated with both histone acetyltransferase that can acetylate HeLa cell-derived nucleosomes on histone H4 and histone methytransferase that can support mono-, di-, and tri-methylation of histone H3 on lysine 4 (H3K4me1, H3K4me2, and H3K4me3) in recombinant histone octamers. In contrast, complexes containing Flag-Ash2 (a subunit shared between MLL and SET1-containing complexes) copurified only with HMT activity (Figure 1B). To further investigate the potential interplay between H3K4 methylation and H4 acetylation by Flag-WDR5-containing complexes, we performed combined assays for HAT and HMT, in which reactions contained both the acetyl group donor acetyl CoA (AcCoA) and the methyl group donor S-adenosyl methionine (SAM). Although the HAT activity associated with Flag-WDR5 complex was not affected in the presence of SAM (S-adenosyl methionine, a methyl donor) (Figure 1C), HMT activity was dramatically increased in the presence of AcCoA-dependent manner (Figure 1D).

### hMOF-mediated acetylation accounts for the positive regulation of H3K4 methylation by MLL/SET complexes

**Figure 2:**
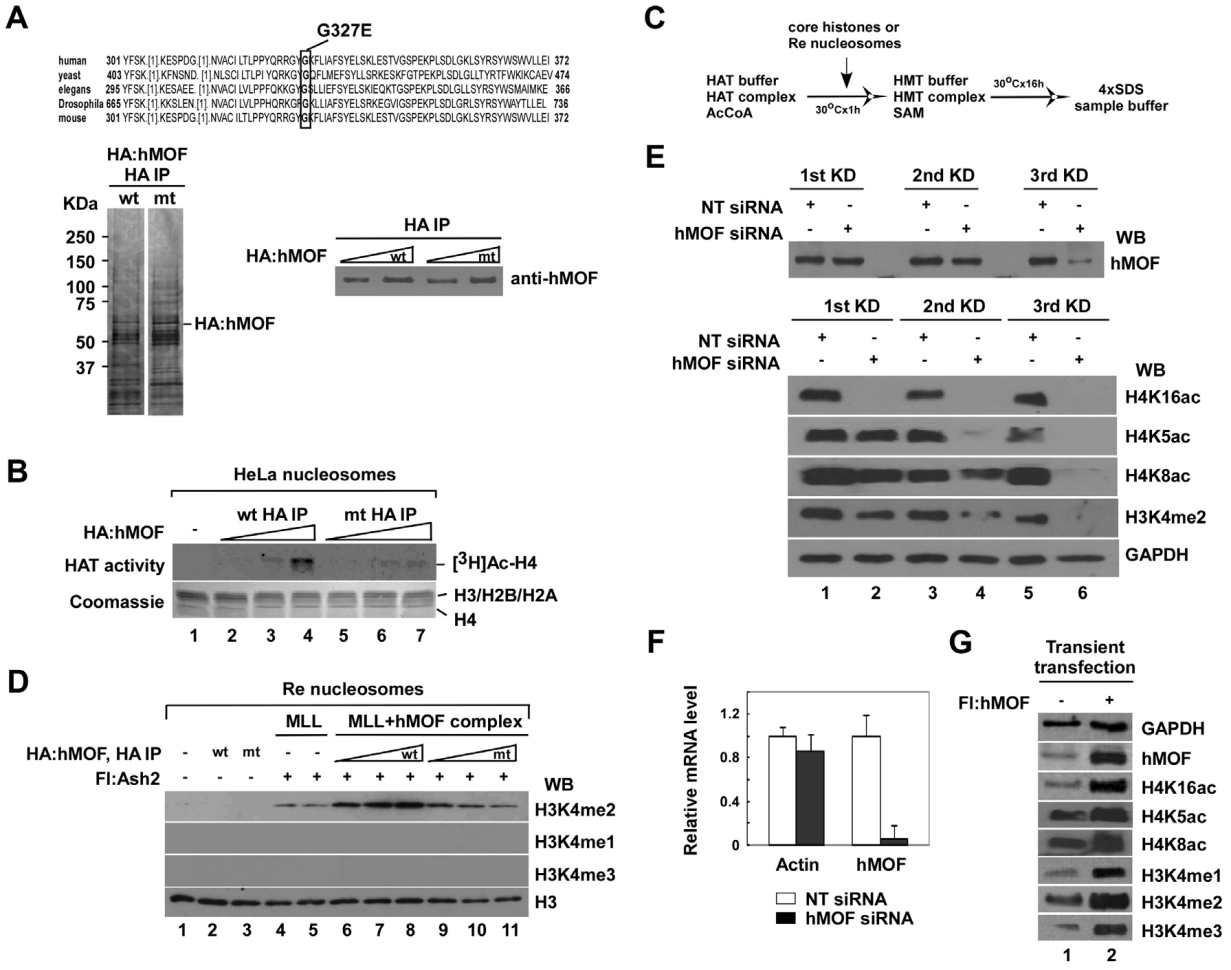
The activity of histone H3K4 methylation is facilitated by hMOF-containing complexes both *in vitro* and *in vivo*. **A.** Purification of hMOFwt- and hMOFmt-containing complexes. HA-purified hMOFwt- or G327E-cantaining complexes were analyzed by silver stain (left) and Western blot (right). **B.** hMOFmt-containing complex lost HAT activity. Relatively equal amounts of hMOF protein in the HA-purified hMOFwt (lane 2–4) or G327E (lane 5–7) complexes were subjected to a HAT assay. Histone proteins were visualized by Coomassie R-250 blue stain (bottom) and radioactively labeled proteins were visualized by autoradiography (top). **C.** Schematic flowchart of the HMT assay *in vitro*. **D.** The activity of H3K4me2 is facilitated by the hMOFwt-containing complex on recombinant polynucleosomes (lane 6–8). The HMT assay was carried out by mixing in the combinations indicated in the figures. Modified residues at H3K4 were detected with methylation-specific antibodies. **E.** Global H3K4me2 is reduced in hMOF siRNA knockdown HeLa cells. HeLa cells were transfected with 20 nM hMOF siRNA and non-targeting siRNA (as control) three times every 48 hours. Then, 48 hours after each transfection, cells were harvested for Western blot analysis and qRT-PCR check (**F**). hMOF protein and modified residues at histone H4 and H3 were detected with indicated antibodies. **G.** Global histone modifications in hMOF overexpressed 293T cells. 293T cells were transiently transfected with 2 µg hMOF cDNAs. Then, 48 hours after transfection, cells were harvested and lysed by adding 4×SDS sample buffer. Proteins were identified with Western blot.

To determine whether the AcCoA-dependent increase in H3K4 methylation activity associated with Flag-WDR5-containing complexes is mediated by hMOF complex(es), we generated HA-tagged hMOF containing a point mutation, G327E, in a highly conserved residue in the hMOF HAT domain (Figure 2A, top) [30]. Complexes containing wild type or mutant HA-tagged MOF purified using anti-HA agarose immunoaffinity chromatography from 293FRT cell lines stably expressing HA-hMOF wild type (hMOFwt) or HA-hMOF G327E (hMOFmt) and fractionated them by SDS-PAGE. As shown in Figure 2A (left), a similar set of polypeptides could be detected in both hMOFwt and hMOFmt complexes by silver staining. To determine how the G327E mutation affects hMOF activity, HA-hMOFwt and HA-hMOFmt complexes containing equivalent amounts of hMOF (Figure 2A, right) were subjected to HAT assays. The results of these experiments indicated that the HAT activity associated with HA-hMOFmt was dramatically reduced (Figure 2B, lane 5–7) compared to HA-hMOFwt (Figure 2B, lane 2–4). To test the role of hMOF-dependent HAT in enhancement of H3K4 methylation, HMT assays were performed according to experimental procedures shown in Figure 2C. In line with the HAT activity, only complexes containing hMOFwt were able to stimulate MLL/SET-mediated H3K4 methylation (Figure 2D, lane 6–8).

*In vitro* experimental results clearly show that hMOF-mediated acetylation accounts for the positive regulation of H3K4 methylation by MLL/SET complexes. To clarify whether the hMOF-mediated HATs also affect global H3K4 methylation in human cells, we knocked down or overexpressed hMOF, which, as noted above, is the catalytic subunit of both MSL and NSL complexes [16]–[18]. In RNA interference (RNAi) experiments, HeLa cells were transfected with hMOF siRNA three times at 48-hour intervals. After 48 hours, the level of hMOF mRNA was significantly decreased compared to non-targeting (NT) siRNA control (Figure 2F), while the level of hMOF protein was not significantly decreased after the first round of siRNA transfection and gradually decreased thereafter (Figure 2E, top). Global acetylation of H4K16 in cells completely disappeared after the first round of hMOF siRNA transfection, consistent with previous reports that hMOF, but not Tip60, is the major HAT responsible for H4K16 acetylation [16], [31]. We also observed that global acetylation of histone H4K5 and H4K8 were substantially reduced after the second and third rounds of hMOF knockdown (Figure 2E, bottom), consistent with our evidence that the hMOF-containing NSL complex supports H4K5 and H4K8 acetylation *in vitro* and suggesting that a hMOF-containing complex is also responsible for acetylation of histone H4K5 and H4K8 in human cells [17]. Notably, we also observed that knockdown of hMOF led to a reduction of global histone H3K4 di-methylation in cells. In contrast to the results of our knockdown experiments, hMOF overexpression promoted the global H4K16, K5, and K8 acetylation as well as H3K4 mono-, di- and tri-methylation (Figure 2G).

### HAT NSL complex, but not MSL, contributes to H3K4me2 by MLL/SET complexes *in vitro*

**Figure 3:**
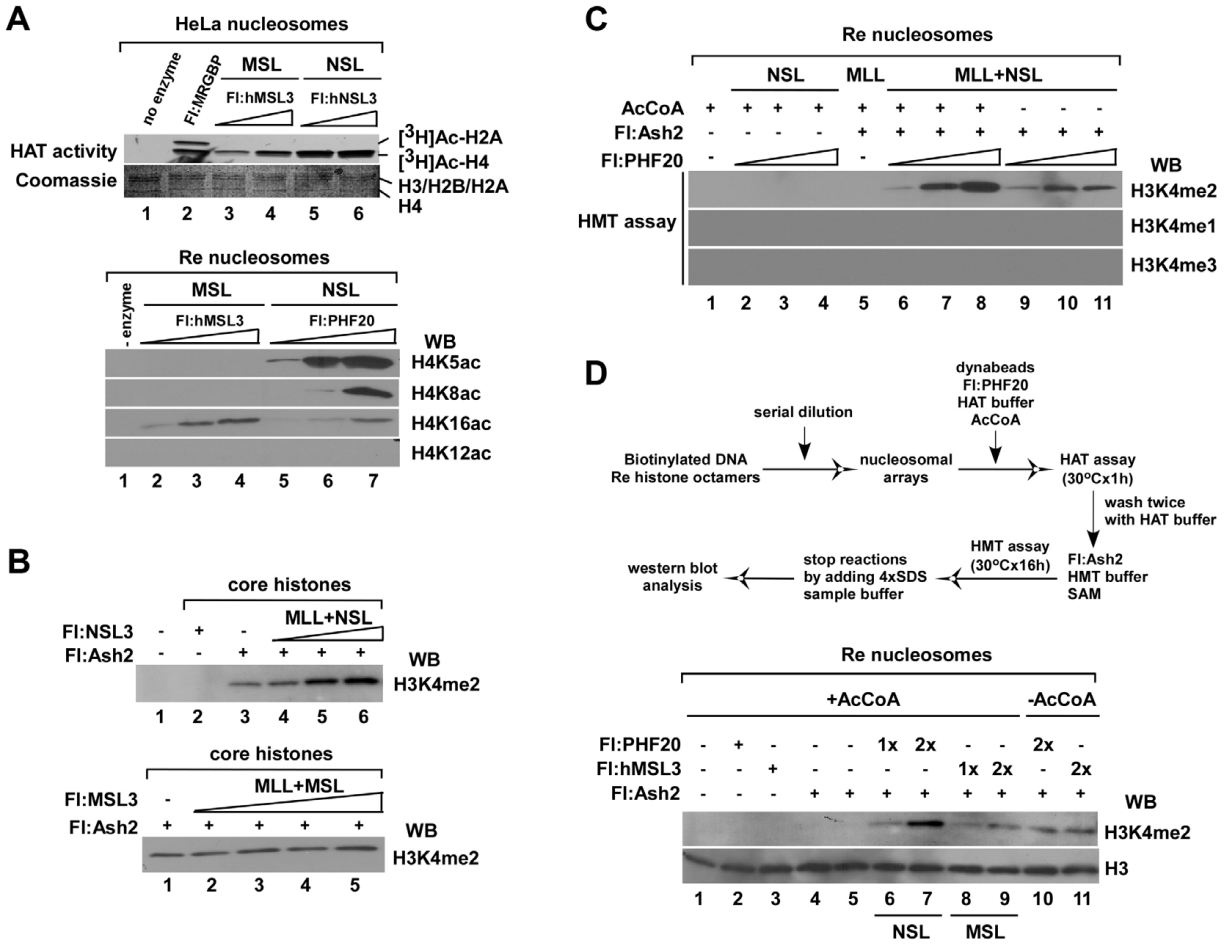
The HAT NSL complex, but not the MSL complex, promotes H3K4me2 by Flag-Ash2 *in vitro*. **A.** Substrate specificity of two hMOF-containing complexes on recombinant polynucleosomes. HAT assays were performed by mixing [^3^H]-labeled Acetyl CoA or cold AcCoA, HeLa nucleosomes or recombinant polynucleosomes and 1–4 µl of the indicated anti-Flag agarose eluates. Histones were visualized with Coomassie R250 blue stain and acetylated histones were visualized by autoradiography (top). Modified residues at histone H4 were detected with acetylation-specific antibodies (bottom). **B–C.** The activity of H3K4me2 by Flag-Ash2 complex is facilitated by HAT NSL, but not the MSL complex *in vitro*. **D.** The NSL complex is required for promoting H3K4me2 activity via the Flag-Ash2 complex on recombinant polynucleosomes. The experimental procedures are as shown at the top. In this experiment, reconstituted nucleosomes were prepared by mixing ∼1500 bp biotinylated oligonucleotides and recombinant human histones through serial dilution. Reconstituted nucleosomes were then immobilized on avidin-coupled Dyna-beads. HAT assays were first performed on immobilized nucleosomes, and the HMT assays were carried out after two washes with HAT buffer. Modified residues at histone H3K4 were detected with H3K4me2 antibody.

It has been reported that hMOF, as a catalytic subunit, forms MSL and NSL, which are two distinct human cellular complexes [16]–[18]. According to the description above, acetylation-methylation interaction occurs in both WDR5- and hMOF-containing complexes. Thus, we speculate that the NSL complex is important in this interaction. To verify this, NSL, MSL and MLL/SET complexes were subjected to a series of *in vitro* HMT assays. We first measured the HAT activities of two complexes purified from stably expressing Flag-tagged indicated proteins. Consistent with our previous reports [17], the MSL complex acetylated histones specifically at the H4K16 site whereas the NSL complex acetylated histone H4 at K5/K8/K16 sites, suggesting a relaxed specificity (Figure 3A). In a combined enzyme activity assay with core histones or recombinant nucleosomes as substrates, the NSL complex facilitated MLL/SET-meditated H3K4me2 in a dose-dependent manner (Figure 3B, 3C). However, the MSL complex did not obviously enhance H3K4me2 in the same assay (Figure 3B). The experimental procedures for Figure 3B and 3C is depicted in Figure 2C. Considering that NSL complex proteins may affect the subsequent HMT assay, reconstituted nucleosomal arrays were prepared and used in combined enzyme activity assay. A scheme of the assay is shown in Figure 3D (top). After removing AcCoA and HATs, the role of promoting H3K4me2 activity by Flag-Ash2 can still be observed in the lane for which the initial HAT assay was performed with the NSL, but not the MSL complex (Figure 3D, bottom). This suggests that the NSL complex—with respect to acetylation of histone H4 specific lysine—might be important for promoting subsequent di-methylation of histone H3K4 via the Flag-Ash2 complex.

### Regulation of gene expression by cooperation of NSL-MLL/SET complexes

**Figure 4:**
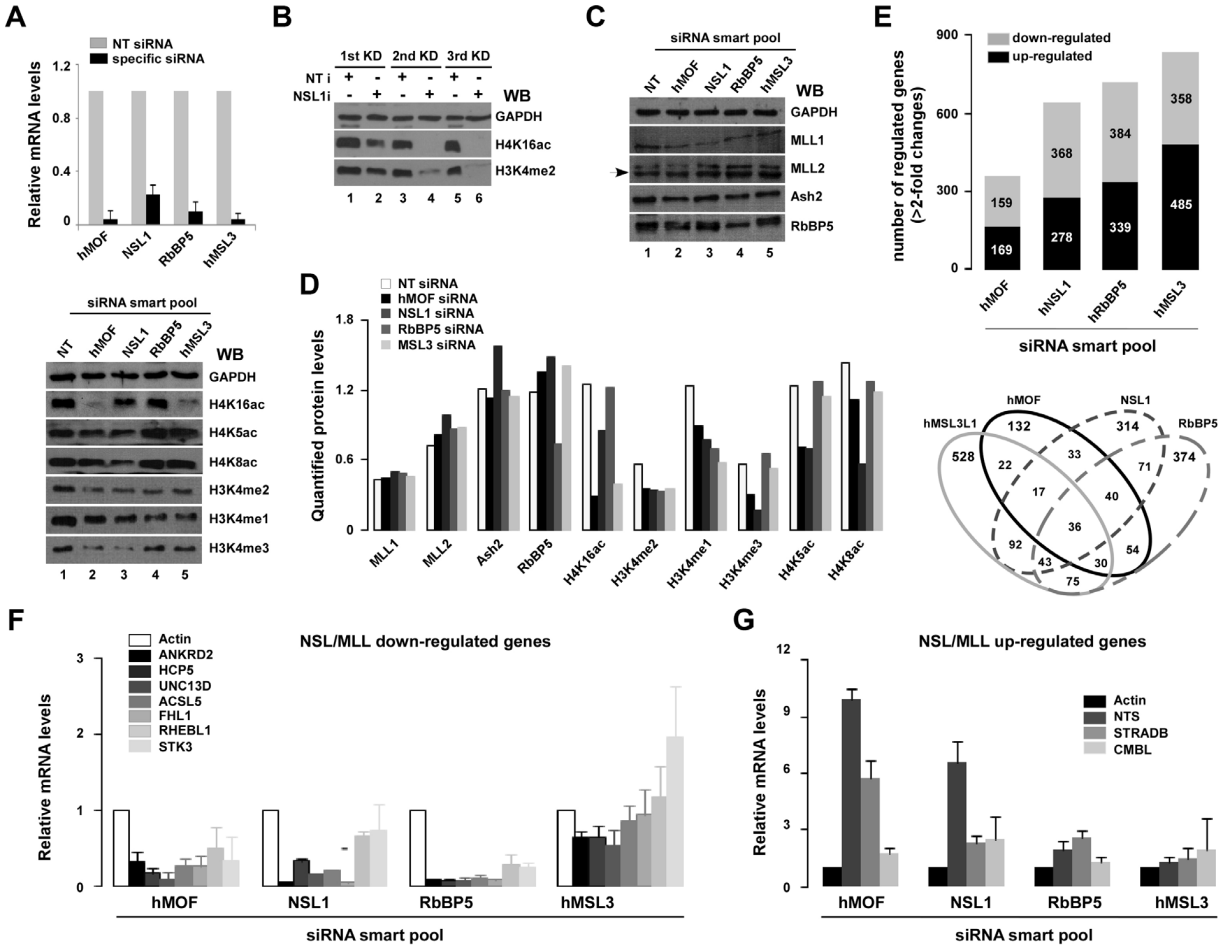
Select genes are co-regulated by NSL and RbBP5-MLL complexes. **A.** Alteration of global histone H4 or H3K4 modifications in hMOF, hNSL1, hRbBP5 or hMSL3L1 siRNA knockdown HeLa cells. HeLa cells were transfected with indicated siRNAs (non-targeting siRNA as control). Next, 48 hours after transfection, cells were lysed and total RNA was isolated with Trizol. mRNA of specific genes were measured with qRT-PCR (top). Whole-cell extracts were prepared 48 hours after transfection with specific-siRNAs and were subjected to SDS-PAGE (18% gel). Modified residues at histone H3K4 or H4K5/8/K16 were detected with specific-modified antibodies (bottom). **B.** Reduction of global H3K4me2 was observed in NSL1 siRNA knockdown HeLa cells. HeLa cells were transfected with 20 nM NSL1 siRNA three times every 48 hours, after which cells were harvested for Western blot analysis. **C.** Protein of subunits of MLL/SET complexes did not change in hMOF or NSL1 siRNA knockdown HeLa cells. HeLa cells were transfected with 20 nM indicated siRNAs. Then, 48 hours after transfection, whole-cell extracts were prepared and proteins were detected using the indicated antibodies. **D.** Quantified protein. Western blot images were scanned and signals were quantified with densitometry using Quantity One Basic software (Bio-Rad). **E.** Gene expression profiles in siRNA knockdown HeLa cells. Stacked column charts represents the total number of regulated genes (>2-fold decrease or increase) in the indicated specific siRNA knockdown HeLa cells. Each bar in the stacked column chart is composed of down- and up-regulated genes (top figure). The Venn diagram depicts shared and distinct gene expression in each population (bottom figure). **F–G.** mRNA of select genes is regulated by NSL and RbBP5-MLL complexes. Genes indicated in figures were measured by qRT-PCR in specific-siRNA knockdown HeLa cells. Bar graphs show ratios of qRT-PCR signals (normalized to non-targeting siRNA) to actin (also normalized to non-targeting siRNA). Error bars represent the standard error of the mean of 3 independent experiments.

Undoubtedly, the HAT NSL complex contributes to H3K4me2 by MLL/SET complexes *in vitro.* To investigate whether potential target genes are specifically regulated via NSL-MLL/SET co-operativity, gene expression was measured after knocking down the core component of the NSL or MLL/SET complexes. Specific siRNA used to knockdown corresponding component in the complex led to significant reductions of specific mRNA (Figure 4A, top). Global histone modifications were then detected after specific siRNA knockdown (Figure 4A bottom & 4B). In agreement with data from the *in vitro* assay, knocking down RbBP5, a protein required for the H3K4 methylation by MLL/SET complexes, significant decreased H3K4me2 but had less effect on cellular H4K16ac, in contrast, disruption of hMOF-mediated H4 acetylation, including H4K16, K5 or K8, by knockdown of hMOF or NSL1 led to various degrees of H3K4 methylation depression. Knocking down hMSL3, a protein required for H4K16ac of the MSL complex, only significantly decreased H4K16ac, but not H4K5ac or H4K8ac (Figure 4A, bottom). In addition, as shown in Figure 4C, knocking down the hMOF or NSL1 gene did not affect the protein levels of subunits of MLL/SET complex. Quantified protein is shown in Figure 4D.

To understand variations in gene transcription among hMOF-mediated and MLL/SET complexes, HeLa cells with specific siRNA knocked down were sent to Phalanx Biotech Group, Inc. for gene expression profile analyses. From DNA microarray analyses using Phalanx Human OneArray (HOA 5.2), a total (>2-fold changes of downregulated and upregulated) of 364, 646, 723 and 843 genes were shown to be differentially expressed among hMOF, NSL1, RbBP5 or MSL3L1 and NT siRNA knockdown HeLa cells, respectively (Figure 4E, top). The shared and distinct gene expression in each population is depicted in a Venn diagram (Figure 4E, bottom). Interestingly, 54 genes were co-regulated by hMOF/RbBP5; 40 genes were co-regulated by hMOF/NSL1/RbBP5; and 30 genes were co-regulated by hMOF/MSL3L1/RbBP5. mRNA from different siRNA knockdown cells was measured with qRT-PCR. As shown in Figure 4F and 4G, compared to internal control actin, selected gene mRNA including ANKRD2, HCP5, UNC13D, ACSL5, FHL1, RHEBL1 and STK3 was downregulated with siRNA knockdown of hMOF, hNSL1, and hRbBP5 in HeLa cells. In contrast, NTS, STRADB and CMBL mRNAs was upregulated. However, no obvious changes were observed for those genes in hMSL3L1 siRNA knockdown cells. Of note, our gene expression profile data of hMOF depleted HeLa cells was similar to that from hMOF-depleted HEK293 cells as reported by Sharma and colleagues [12]. This indicates conserved transcriptional regulation of hMOF among different cell lines.

### NSL complex is essential for targeting of MLL/SET complexes to ANKRD2

**Figure 5:**
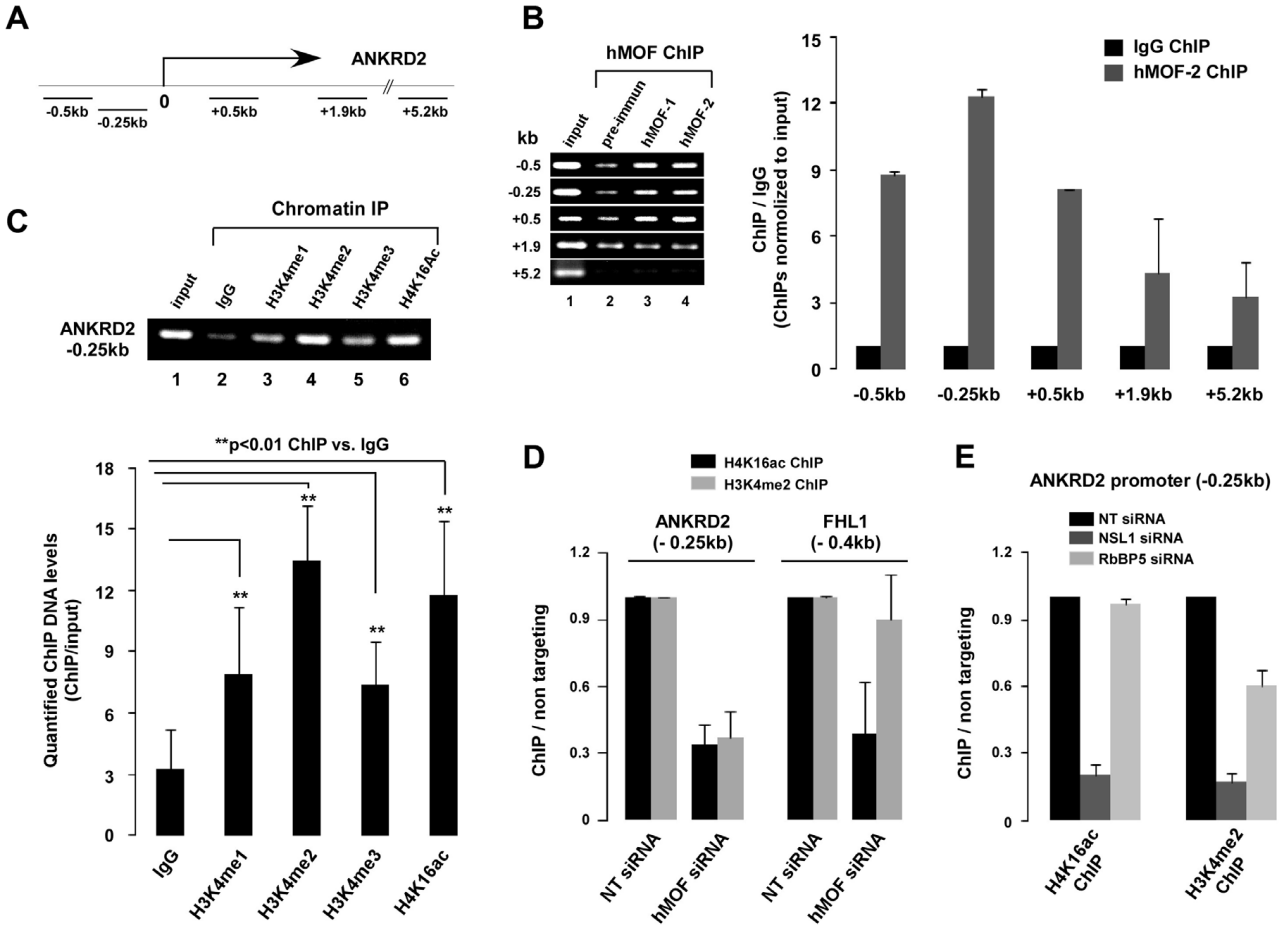
NSL complex is essential for targeting of RbBP5-MLL complex to ANKRD2. **A.** Five primer sets in the ANKRD2 locus used for amplifying ChIP'd DNA. **B.** Distribution of hMOF on the ANKRD2 locus. ChIP assays were performed using hMOF antibody. ChIP'd DNA was analyzed by qPCR. Bar graph shows the ratios of ChIP'd DNA signals (normalized to input) to IgG (also normalized to input; right panel). Error bars represent the standard error of the mean of 3 independent experiments. Significant. **C.** Enrichment of histone H3K4me and H4K16ac on the ANKRD2 promoter region (−0.25 kb). ChIP assays were performed using H3K4me1/me2/me3 or H4K16ac antibodies. ChIP'd DNA was amplified by PCR (top). Quantified PCR signals (Quantity One software) were analyzed by *t*-test (bottom). Error bars indicate SE and the significant difference is expressed as **p<0.01. **D.** Functional cooperation of histone H4K16ac and H3K4me2 on ANKRD2 (−0.25 kb), but not on FHL1 (0.4 kb) gene promoter. ChIP assays using hMOF siRNA knockdown HeLa cells were analyzed by qPCR. **E.** The NSL complex is essential for binding of the RbBP5-MLL complex to the ANKRD2 gene promoter region (−0.25 kb). ChIP assays using hNSL1 and hRbBP5 siRNA knockdown HeLa cells were analyzed by qPCR. Bar graphs show ratios of ChIP signals (also normalized to input DNA and IgG) to non-targeting siRNA controls (normalized to input DNA and IgG). Error bars represent the standard error of the mean of 3 independent experiments.

The previously mentioned, our results suggest that the expression of some genes may require coordinated regulation of the NSL-MLL/SET complexes. Therefore, the *ANKRD2* gene was chosen for investigating the role of coordinated regulation of the NSL-MLL/SET complexes in gene transcription. Five primer sets were designed to yield ChIP'd DNA for the ANKRD2 promoter proximal region as well as the region far away from the transcriptional start site (Figure 5A). Analysis of ChIP assays for HeLa cells revealed that the distribution of hMOF was restricted to the ANKRD2 transcriptional start site proximal region (from −0.5 kb to +0.5 kb) (Figure 5B), which was in agreement with H4K16ac distribution reported in downregulated genes in hMOF depleted HEK293 cells [20]. To illustrate the correlation between hMOF/H4K16ac and H3K4me2 on the ANKRD2 transcriptional start site region, ChIP assay was performed using H4K16ac, H3K4me1, H3K4me2 and H3K4me3 antibodies. As shown in Figure 5C, marks of H4K16ac and H3K4 methylation, especially H3K4me2, were enriched −0.25kb upstream of the transcription start site, representing the colocalization of H4K16ac and H3K4me with hMOF on the ANKRD2 promoter region.

The correlation between hMOF/H4K16ac and H3K4me2 on the ANKRD2 promoter prompted us to examine transcriptional regulation of ANKRD2 via cooperation of the NSL-MLL/SET complexes. For this, specific genes including hMOF, NSL1, or RbBP5 were depleted by siRNAs in HeLa cells followed by ChIP assays for H4K16ac and H3K4me2 at the ANKRD2 transcriptional start site-proximal region (−0.25 kb). As shown in Figure 5D, depletion of hMOF significantly reduced both H4K16ac and H3K4me2 around the ANKRD2 transcriptional start site proximal region. Around the FHL1 promoter region (−0.4 kb), depletion of hMOF only decreased H4K16ac marks, but not H3K4me2. Similarly, depletion of NSL1 significantly reduced both H4K16ac and H3K4me2 around the ANKRD2 transcriptional start site proximal region. However, depletion of RbBP5 only reduced H3K4me2 marks, not H4K16ac, in the same region of ANKRD2, suggesting that ANKRD2 gene transcription is co-regulated by NSL-MLL/SET complexes (Figure 5E). In addition, distribution of H3K4me2 at the ANKRD2 promoter proximal region was affected by hMOF or NSL1 siRNA-induced H4K16ac reduction.

## Discussion

In this report we confirmed the existence of functional cooperation between hMOF-containing NSL and MLL/SET complexes using both *in vitro* and *in vivo* approaches. HAT-HMT combined *in vitro* assays present evidence that hMOF-containing NSL complex functions to promote histone H3K4 methylation via MLL/SET complexes. Analysis of gene expression indicates that the expression of some genes is coordinated by the NSL and MLL/SET complexes. In addition, coordination between NSL and MLL/SET complexes is involved in transcriptional regulation of certain genes, such as ANKRD2.

Histone H4K16ac and H3K4me are critical in mammalian cells and function as specific transcription regulators that directly linked to either gene transcription activation or repression [18], [27]–[28]. Although the coordinated activity of H4K16ac and H3K4me has been observed in transcription regulation of certain genes, such as HOX and FOXP3-activated genes [29], the precise crosstalk mechanism remains unclear. Based on *in vitro* HAT and HMT assays with distinct enzymatic complexes purified from stably expressed Flag-tagged proteins, we defined a positive regulation of MLL/SET-mediated H3K4 methylation by Flag-WDR5 complex in AcCoA-dependent manner. Further investigation revealed that enhanced activity of H3K4me by MLL/SET complexes was indeed due to the hMOF-containing complex; no similar enhancement was observed with induction of a G327 point mutation in hMOF. To support this observation, reduction of global H3K4me in cells was confirmed by knocking down hMOF or NSL1 with siRNAs, suggesting that the hMOF/NSL complex may be involved in cellular H3K4 methylation regulation.

Although both the NSL and the MSL HAT complexes contain hMOF as a catalytic subunit, assembly of hMOF HAT into the MSL or NSL complex controls its substrate specificity and transcription regulation. NSL-associated hMOF has less specificity for nucleosomal histone H4 [17] and appears to be involved in more global regulation of transcription [21]–[23]. In our RNAi experiments, knocking down hMOF or NSL1 not only decreased H4K16ac, but also reduced cellular H4K5ac and H4K8ac (Figure 2E, 4A & 4B). In contrast, in hMSL3 knockdown HeLa cells, no decline in H4K5ac or H4K8ac was observed, except for the reduction of H4K16ac. Our data show that the NSL complex, but not the MSL complex, plays a role in promoting histone H3K4me2 activity by MLL/SET complexes (Figure 3B–D), indicating the functional differences between the two complexes. An attractive hypothesis is that acetylation of histone H4 on K5, K8, and/or K16 might be responsible for this effect; however, we cannot rule out the possibility that there were small/trace amounts of residue NSL complex remaining to acetylate some component(s) of the MLL/SET complex. However, knocking down either NSL1 (a core component of the NSL complex) or MSL3 (a core component of the MSL complex) to disrupt the NSL or MSL complex, respectively [32]–[33], suppressed global activity of both cellular H4K16ac and H3K4me. These data indicate that more a complicated regulatory pathway may be involved in the MSL complex in the cellular environment. Recent studies identified that the MSL2 protein in the MOF complex is an E3 ubiquitin ligase for H2BK34 involved in crosstalk with H3K4 and K79 methylation [34]. In addition, when using recombinant nucleosomes, a chromatin structural form more close to native status than to histone octamers, only MLL/SET-mediated H3K4me2 can be detected *in vitro* (Figure 2D, 3C & 3D). In contrast, knockdown or overexpression of cellular hMOF reveals alterations in all methylation scales (Figure 2E, 2G & 4A). Although only one H3K4 methyltransferase exists in yeast to catalyze H3K4 mono-, di-, or tri- methylation, mammalian cells contain at least 10 H3K4 methyltransferases with various specificities *in vivo* [35]–[36]. Thus H3K4 methylation is more complicated *in vivo* than *in vitro*. So, MLL/SET- mediated H3K4me2 detected *in vitro* may be more reliable and probably acts as a node of the NSL-MLL/SET cooperative network. However, we cannot exclude the possibility of differences in antibody titers.

Gene expression studies combined with siRNA knockdown data provides rich information about gene transcription regulated by the NSL, MLL/SET and MSL complexes. First, after specific siRNA knockdown, in a core component of the above mentioned three complexes, transcriptions of 2576 genes were found to be altered more than two-fold. Among these genes, 513 genes overlapped in 2 or more knockdown samples, suggesting a strong correlation between histone modifications and gene expression regulation. Next, 160 genes were co-regulated by hMOF and RbBP5 knockdown, suggesting collaborative roles between hMOF-mediated HAT activity and MLL/SET-mediated HMT activity. Finally, among the 160 genes co-regulated by the hMOF and MLL/SET complexes, 40 genes are affected by NSL1 only, whereas 30 genes are influenced by MSL3L1 only. This suggests distinct functions of the NSL and MSL complexes in hMOF-MLL/SET coordination.

It was previously reported that hMOF is essential for embryonic development [11], [37]. It is worth noting that our gene expression profile analyses identified genes co-regulated by hMOF/NSL1/RbBP5 that were related to tissue and organ formation and occurrence of various diseases. ANKRD2, a muscle ankyrin repeat protein, is important in muscle gene transcription regulation, myofibrillar assembly, cardiogenesis, and myogenesis. Abnormal expression of the *ANKRD2* gene leads to neuromuscular disorders, cardiovascular diseases, and even cancer [38]. ChIP assay with available antibodies revealed detailed collaborative mechanism of hMOF/NSL1/RbBP5 on ANKRD2 gene expression regulation. Distribution of hMOF, H4K16ac and H3K4me2 were restricted to the ANKRD2 transcriptional start site proximal region. H4K16ac and H3K4me2 are mediated by hMOF/NSL HAT and MLL/SET HMT: knockdown of the core subunit of these complexes significantly blocks corresponding histone modifications (Figure 5D & 5E). Notably, hMOF/NSL-mediated H4K16ac promotes MLL/SET-mediated H3K4me2, a finding that is in agreement with data obtained from *in vitro* enzyme assays (Figure 5E). In summary, coordination between hMOF/NSL-mediated H4K16ac and MLL/SET-mediated H3K4me2 is involved in ANKRD2 gene activation. Consistent with our data, we noted that NSL-bound genes exhibit elevated H3K4me2/3, H3K9ac and H4K16ac according to genome-wild analyses in *Drosophila* [23]. This may be another hMOF/NSL-MLL/SET correlation instance that is similar to our findings in mammalian cells.

In our study, the regulatory effect of hMOF/NSL HAT to MLL/SET HMT activity seemed to be unidirectional: no obvious reverse effects from methylation to acetylation were detected in either *in vitro* assays or endocellular circumstances. These data indicate that hMOF/NSL-dependent acetylation event(s) regulate MLL/SET activity; thus, the pathway is unidirectional. However, some questions persist about this cooperation: 1) Does hMOF/NSL-mediated acetylation of histone H4 induce alteration of chromatin structure and facilitate the subsequent methylation process? 2) Does the NSL complex help with recruiting and assembling of MLL/SET HMT complexes at upstream regions of target genes? In addition, it is important to determine which components of the NSL complex play synergistic roles in the regulation of H3K4 methylation via MLL/SET complexes. Additionally, MLL is a proto-oncogene that is rearranged in a wide variety of human leukemias, so future studies are warranted to investigate whether the NSL complex is implicated in MLL-rearranged leukemia.

## Materials and Methods

### Materials

Anti-H4K16ac (H9164) antibody was obtained from Sigma (U.S.A.). Anti-H3K4me1 (07-436), anti-H3K4me2 (07-030) and anti-H3K4me3 (07-473), anti-H3 (06-755), anti-H4K5ac (07-327), anti-H4K8ac (07-328) and anti-WDR5 (07-706) rabbit polyclonal antibodies were from Millipore (U.S.A.). Anti-OGT1 (sc-32921) and anti-MLL1 (sc-18214) antibodies and rabbit total IgG (sc-2027) were purchased from Santa Cruz Biotechnology (U.S.A.). Anti-MCRS1, anti-hMOF (residue 210–468 aa), anti-RbBP5, anti-Ash2, anti-MSL3L1 and anti-GAPDH rabbit polyclonal antibodies were raised against bacterially expressed proteins (Jilin University). Anti-MSL2L1 (H00055167A01) was from Novus Biologicals. Anti-MSL1 antiserum was kindly provided by Dr. Edwin R. Smith (Stowers Institute for Medical Research).

### Generation and growth of mammalian cell lines

Full-legth cDNAs encoding the human WDR5 (NM_017588), NSL2 (FLJ20436; BC009746), NSL3 (FLJ10081; BC063792), PHF20 (NM_016436), hMSL3 (male-specific lethal 3-like 1; BC031210) and hAsh2 ((absent, small, or homeotic)-like; NM_004674) proteins were obtained from the American Type Culture Collection (ATCC), subcloned with FLAG tags into pcDNA5/FRT, and introduced into HEK293/FRT cells using the Invitrogen Flp-in system [17]. hMOF cDNAs (wild type or mutant G327E) were subcloned with HA-tag into pQC and introduced into 293FRT cells. Stably transformed HEK293/FRT cells were maintained in Dulbecco's modified Eagle's medium (Sigma) with 5% glucose and 10% fetal bovine serum.

### Immunoaffinity purification of protein complexes

Nuclear extracts were prepared from HEK293/FRT cells according to the method of Dignam *et al.* [39]. Flag-tagged or HA-tagged proteins and their associated proteins were purified on anti-Flag (M2) or anti-HA agarose beads as previously described [40]. Identification of proteins was accomplished using a modification of the MudPIT (multidimensional protein identification technology) procedure described by Washburn *et al.* [41].

### Preparation of the histone octamers and polynucleosomes

Recombinant histone octamers and polynucleosomes (used ∼1500 bp DNA fragment) were prepared as previously described [40], [17].

### Histone acetyltransferase (HAT) assay

Histone acetyltransferase assays were performed essentially as described [17].

### Histone methyltransferase (HMT) assay

Reaction mixtures (32 µl) containing 50 mM Tris-HCl (pH 8.5), 20 mM KCl, 10 mM MgCl_2_, 250 mM sucrose, 10 mM β-mercaptoethanol, 1 mM protease inhibitor cocktail (Sigma), 125 µM S-adnosyl methionine (SAM, Sigma), 0.5 µg *E.coli* expressed and purified core histones, or 2 µg of long oligo-nucleosomes or reconstituted polynucleosomes, and anti-Flag or HA-agarose eluates prepared from HEK293/FRT cells stably expressing Flag-tagged or HA-tagged proteins were incubated at 30°C for 16–18 hours. Reactions were stopped by addition of 4×SDS sample buffer and was then fractionated on 18% SDS-PAGE and subjected to Western blotting using methylation-specific antibodies to detect modified histone H3 residues.

### RNAi treatment and DNA microarray

HeLa cells were cultured in 6-well tissue culture plates (∼2×10^5^ cells/well) in DMEM medium (Sigma) containing 10% fetal bovine serum. The cells were transfected with 20 nM non-targeting siRNA (D-001206), MYST1 siRNA (D-014800), KIAA1267 siRNA (D-031748), MSL3L1 siRNA (D-012319) and RbBP5 siRNA (D-012008) SMART pool (Dharmacon, U.S.A.). Then, 24 hours after transfection, cells were divided into new 6-well plates for immunoblotting, RT-PCR and DNA microarray analysis. Finally, 24 hours later, cells were harvested and lysed. Whole-cell extracts were prepared from cells from 3/4 of the wells in a 6-well plate by adding 4× SDS sample buffer, and total RNA was isolated from 1/4 of the wells in a 6-well plate using TRIzol LS Reagent (Invitrogen). In addition, cells from 1 well of a 6-well plate were rinsed twice with warm PBS and harvested. Cells were then stored in an RNA hold solution (ER501-01, Beijing Transgen Biotech Co., Ltd.) and sent to OneArray by Phalanx Biotech Group for DNA microarray analysis.

### Transient transfection

Human embryonic kidney (HEK) 293T cells were cultured in 6-well tissue culture plates (∼2×10^5^ cells/well) in DMEM containing 10% fetal bovine serum and antibiotics. The cells were transiently transfected with 2 µg of hMOF cDNAs using Lipofetamine 2000 (Invitrogen). At 48 hrs post-transfection, cells were harvested and lysed by adding 4×SDS sample buffer. Whole-cell extracts were analyzed by Western blotting with the indicated antibodies.

### Reverse transcription PCR (RT-PCR)

Cells from 1 well of a 6-well plate were lysed and total RNA was isolated using Trizol. Total RNA (1 µg) from each sample was used as a template to produce cDNA with PrimeScript 1st Strand cDNA Synthesis Kit (TAKARA). MYST1, NSL1, RbBP5, MSL3L1 and GAPDH mRNA was measured by quantitative real time PCR with Real Time PCR Detector Chromo 4 (Bio-Rad). All PCR reactions were finished under the following program: initial denaturation step was 95°C for 3 min, followed by 35 cycles of denaturation at 95°C for 30 seconds, annealing at 60°C for 30 seconds and extension at 72°C for 30 seconds. The following qRT-PCR primer sets were used to verify the siRNA knockdown efficiency: hMOF, 5′-GGCTGGACGAGTGGGTAGACAA-3′ (forward) and 5′-TGGTGATCGCCTCATGCTCCTT-3′ (reverse), yielding a 227 bp product; hNSL1, 5′-CTTATTGCTGCCAACGGAACCA-3′ (forward) and 5′-AGGACTGTCTGCTTGCTGAAGA-3′ (reverse), yielding a 196 bp product; hMSL3L1, 5′-CAGGACACATCCGCCAGCAT-3′ (forword) and 5′-AAAGCCAGCAAACACAGCACTC-3′ (reverse), yielding a 128 bp product; hRbBP5, 5′-ATGAACCTCGAGTTGCTGGA-3′ (forword) and 5′-CACTGGATGGATGTGTGCAC-3′ (reverse), yielding a 207 bp product. The primer sets used for qPCR to verify hMOF or NSL1 or RbBP5-regulated genes were as follows: ANKRD2, 5′-TGGCACAGGAGGAGGAGAATGA-3′ (forword) and 5′-CTTCCGCAGCTCGATGAGGTTC-3′ (reverse), yielding a 215 bp product; HCP5, 5′-GACTCTCCTACTGGTGCTTGGT-3 (Forward) and 5′-CACTGCCTGGTGAGCCTGTT-3′ (reverse), yielding a 241 bp product; UNC13D, 5′-GCTGCCACCGTCCTCTTTCT-3′(forword) and 5′-CTCCTCCTGCTGTTCTGCCTTG-3′ (reverse), yielding a 192 bp product; ACSL5, 5′-GCGTCATCTGCTTCACCAGTG-3′ (forword) and 5′-CGTCAGCCAGCAACCGAATATC-3′ (reverse), yielding a 249 bp product; FHL1, 5′-ACTGCGTGACTTGCCATGAGAC-3′ (forword) and 5′-TGGTCCTCCACAGCGGTGAA-3′ (reverse), yielding a 172 bp product; RHEBL1, 5′-CGGGTGCCAGTGGTTCTAGT-3′ (forword) and 5′-CGACGCTCTTGCCCATAGGAA-3′ (reverse), yielding a 206 bp product; STK3, 5′-ATGCGGGCCACAAGCACGAT-3′ (forword) and 5′-TCACCATGGTCCCCAAGTCGGA-3′ (reverse), yielding a 91 bp product; NTS, 5′-GCTCCTGGAGTCTGTGCTCA-3′ (forword) and 5′-CCTTCTTGCAACAAGCTCCTCT-3′ (reverse), yielding a 209 bp product; STRADB, 5′-TGGAGCCGTGAGAGGGTTGA-3′ (forword) and 5′-ACTGATGTGCTGAACTGTGGGA-3′ (reverse), yielding a 189 bp product; CMBL, 5′-GCTAGGCCGTGAAGTTCAAGTC-3′ (forword) and 5′-AAGATAGACCAGTCGCCAGAGG-3′ (reverse), yielding a 222 bp product. The primer sets used for internal control β-Actin were as follows: 5′-ATGGGTCAGAAGGATTCCTATGT-3′ (forword) and 5′-AGCCACACGCAGCTCATT-3′ (reverse), yielding a 153 bp product.

### Chromatin Immunoprecpitation (ChIP)

One or two 10 cm dishes (∼1×10^7^) of HeLa cells grown to ∼80% confluence were used for each ChIP assay. Cells were cross-linked with 5 ml 1% formaldehyde in PBS for 15 min at room temperature followed by incubation with 125 mM glycine for 5 min. To shear DNA to lengths ranging between 200–1000 base pairs, cell lysates were sonicated with a SCIENTZ-IID (Ningbo Xinzhi Biotechnology Co., Ltd., China) for 5×60 seconds with every second interval, at a setting of 45% duty, level 2. Equal amounts of sonicated chromatin from each sample were incubated at 4°C overnight with 5–10 µg of antibodies against H3K4me1, H3K4me2, H3K4me3, H4K16ac, or hMOF-1 rabbit polyclonal antibodies. Total rabbit IgG and pre-immune serum were used as control. The next day, 50 µl of protein A agarose containing salmon sperm DNA (10 µg) and BSA (25 µg) (50% slurry) were added, and the mixture was further incubated for 2.5 hours at 4°C to collect the agarose/antibody/protein complexes. The protein A agarose/antibody/protein complexes were washed for 5 min on a rotating platform with 1 mL buffer in the following order: Low Salt (buffer containing 150 mM NaCl, 0.1% SDS, 1% TritonX-100, 2 mM EDTA and 20 mM Tris, pH8)–High Salt (buffer containing 500 mM NaCl, 0.1% SDS, 1% TritonX-100, 2 mM EDTA and 20 mM Tris, pH8)–250 mM LiCl (buffer containing 0.25 M LiCl, 1% NP-40, 1% NaDOC, 1 mM EDTA and 10 mM Tris, pH8)–TE (buffer containing 10 mM Tris, pH8 and 1 mM EDTA)–TE. Finally the washed beads were eluted with 480 µl elution buffer containing 0.1 M NaHCO3 and 1% SDS. DNA was extracted with phenol/chloroform and precipitated by ethanol. Next, 1–2 µl immunoprecipitated or 100-fold diluted input DNA was amplified with a Real Time PCR Detector Chromo 4 (Bio-Rad). Each experiment was performed 2–3 times independently. All ChIP signals were normalized to total input. The primer sets for qPCR on the promoter region of ANKRD2 were as follows: ANKRD2 −0.5 kb (−262∼−88), 5′- GCAGTTCCCTAGCAGATTAACCT-3′ (forward) and 5′-GCCCAGACAGTGCCAGACTT-3′ (reverse); −0.25 kb, 5′-CTTAACGGGGAAGCATGTGG-3′ (forward) and 5′-GACAGTTCTGTACTCCCAGGCTG-3′ (reverse); +0.5 kb, 5′-GGAGGAGGAGAATGAGGTGC-3′ (forward) and 5′-ACCCCCTGCCAGTAATACCC-3′ (reverse); +1.9 kb, 5′-GTAAGCCGAGATCGCACCAC-3′ (forward) and 5′-AACTTCAGCTCCTGCATTTCC-3′ (reverse); +5.2 kb, 5′-CTGGTGGCCTTTAATGTTGTT-3′ (forward) and 5′-GGTGGTCTCAGAGCCCTTCT-3′ (reverse); ; FHL1 promoter (−0.4 kb), 5′-CGGCTTGCTACTAAGGGGAGG-3′ (forward) and 5′-GCAACAAAGACAGCCAAGTGAGG-3′ (reverse).
